# Complete Genome Sequences of Two Predatory Bacterial Strains, *Bacteriovorax* sp. HI3 and *Myxococcus* sp. MH1, Isolated from a Freshwater Pond

**DOI:** 10.1128/mra.01146-22

**Published:** 2022-11-21

**Authors:** Daisuke Inoue, Naoto Hiroshima, Hidehiro Ishizawa, Hideo Dohra, Michihiko Ike

**Affiliations:** a Division of Sustainable Energy and Environmental Engineering, Osaka University, Suita, Osaka, Japan; b Department of Applied Chemistry, University of Hyogo, Himeji, Hyogo, Japan; c Research Institute of Green Science and Technology, Shizuoka University, Hamamatsu, Shizuoka, Japan; d Department of Science, Graduate School of Integrated Science and Technology, Shizuoka University, Hamamatsu, Shizuoka, Japan; University of Delaware

## Abstract

We report the complete genome sequences of two predatory bacterial strains, *Bacteriovorax* sp. HI3 and *Myxococcus* sp. MH1, which were isolated from a freshwater pond. These two strains are grouped with the *Bdellovibrio* and like organisms and myxobacteria, respectively. Their genomes expand our knowledge of the characteristics of predatory bacteria.

## ANNOUNCEMENT

Predatory bacteria prey on other bacteria for their growth. They play a major ecological role in shaping the microbial community structure and in microbial food webs (1). Their bacteriolytic activities have also gained increasing attention for biotechnological application, mainly as living antibiotics and biocontrol agents (2). However, despite extensive studies on their predation features, genetic information on predatory bacteria remains limited. Here, we report the complete genome sequences of two predatory bacterial strains with different predation strategies, *Bacteriovorax* sp. HI3 and *Myxococcus* sp. MH1, isolated from a freshwater pond in Osaka, Japan, using a double-layer agar plating technique with Escherichia coli HB101 and *Acidovorax* sp. DW036 as the prey, respectively (3).

For DNA extraction from HI3, a segment of an HM buffer-based double-layer agar plate (3) with a lytic halo that appeared following predation of the E. coli strain (prey) by HI3 was picked and cultivated in HM buffer containing the E. coli strain at 28°C with rotary shaking (120 rpm). The resulting culture was filtered through a 0.45-μm filter twice to remove the prey, followed by genomic DNA extraction from the filtrate using a GenElute bacterial genomic DNA kit (Sigma-Aldrich, St. Louis, MO, USA). A single colony of MH1 on LB agar medium (Lennox; Becton, Dickinson and Company, Sparks, MD, USA) was cultivated in LB broth at 28°C with rotary shaking (120 rpm), and genomic DNA was extracted using an illustra bacteria genomicPrep mini spin kit (GE Healthcare, Buckinghamshire, UK). The genomic DNA was sheared using a g-TUBE device (Covaris, Woburn, MA, USA) and size selected using the BluePippin system with a 0.75% agarose gel cassette (Sage Science, Inc., Beverly, MA, USA) for PacBio Sequel II HiFi sequencing (Pacific Biosciences, Menlo Park, CA, USA). SMRTbell templates were prepared according to the manufacturer’s instructions and sequenced at Macrogen, Inc. (Seoul, South Korea). PacBio HiFi reads with lengths of ≥7,500 bp were assembled using Flye v. 2.8.3 (4) (parameters: pacbio-hifi, genome size of 3.8 and 10 Mb for HI3 and MH1, respectively). The resulting single contig was manually rotated using Geneious Prime 2022 (5) to place the *dnaA* gene at the first position of the circular chromosome sequence. The HiFi reads were aligned with the chromosome sequence using pbmm2 v. 1.3.0 (https://github.com/PacificBiosciences/pbmm2), and assembly errors were corrected using Pilon v. 1.23 (6). Gene prediction and annotation were performed with DFAST-core v. 1.2.18 (7) using MetaGeneAnnotator v. 2008/08/19 (8) or GeneMarkS2 v. 1.14_1.25 (9), RNAmmer v. 1.2 (10), and tRNAscan-SE v. 2.0.5 (11) to predict protein-coding sequences (CDSs), rRNAs, and tRNAs, respectively. Default parameters were used for all software unless otherwise specified. Information on the obtained reads and generated genome sequences is summarized in Table 1.

**TABLE 1:** General features of genome sequencing and assembly of *Bacteriovorax* sp. HI3 and *Myxococcus* sp. MH1

Category	Feature	Data for strain:	
		*Bacteriovorax* sp. HI3	*Myxococcus* sp. MH1
HiFi reads	No. of reads	55,792	54,347
	Total bases (bp)	678,127,521	544,628,191
	*N*_50_ (bp)	12,282	10,162
Filtered reads	No. of reads	55,717	54,187
	Total bases (bp)	677,903,128	543,792,599
	*N*_50_ (bp)	12,282	10,165
Assembly	No. of contigs	1	1
	Genome size (bp)	3,858,964	10,778,154
	GC content (%)	40.9	70.2
	Mean coverage (×)	175	50
	No. of CDSs	3,797	8,472
	Coding ratio (%)	93.5	90.5
	No. of rRNAs	6	9
	No. of tRNAs	33	82
Accession data	GenBank accession no.	AP026946	AP026947
	SRA accession no.	DRR413265	DRR413266

The complete genomes of HI3 and MH1 each consist of a single circular chromosome, with sizes characteristic of *Bdellovibrio* and like organisms and myxobacteria, respectively (Table 1). They contain putative genes associated with predation (e.g., various secretion systems and hydrolyzing enzymes, chemotaxis, flagellar assembly, and gliding motility). Further genome analysis will help elucidate the physiology, predation mechanisms, and ecological functions of predatory bacteria.

### Data availability.

The complete genome sequences of HI3 and MH1 have been deposited at DDBJ/ENA/GenBank under accession no. AP026946 and AP026947, respectively. They are linked to BioProject accession no. PRJDB14458, BioSample accession no. SAMD00547686 (HI3) and SAMD00547687 (MH1), and DDBJ Sequence Read Archive accession no. DRR413265 (HI3) and DRR413266 (MH1).
